# Herpes Simplex Virus 2 Counteracts Neurite Outgrowth Repulsion during Infection in a Nerve Growth Factor-Dependent Manner

**DOI:** 10.1128/JVI.01370-20

**Published:** 2020-09-29

**Authors:** Kai A. Kropp, Alberto Domingo López-Muñoz, Birgit Ritter, Rocío Martín, Alberto Rastrojo, Sangar Srivaratharajan, Katinka Döhner, Akshay Dhingra, Julia S. Czechowicz, Claus-Henning Nagel, Beate Sodeik, Antonio Alcami, Abel Viejo-Borbolla

**Affiliations:** a Institute of Virology, Hannover Medical School, Hannover, Germany; b Centro de Biología Molecular Severo Ochoa (Consejo Superior de Investigaciones Científicas and Universidad Autónoma de Madrid), Madrid, Spain; c Heinrich Pette Institute, Leibniz Institute for Experimental Virology, Hamburg, Germany; d Cluster of Excellence RESIST (EXC 2155), Hannover Medical School, Hannover, Germany; University of California, Irvine

**Keywords:** axon guidance, glycoproteins, herpes simplex virus, neuron, neurotrophins

## Abstract

During primary infection, herpes simplex virus 2 (HSV-2) replicates in epithelial cells and enters neurites to infect neurons of the peripheral nervous system. Growth factors and attractive and repulsive directional cues influence neurite outgrowth and neuronal survival. We hypothesized that HSV-2 modulates the activity of such cues to increase neurite outgrowth. To test this hypothesis, we exposed sensory neurons to nerve growth factor (NGF) and mock- or HSV-2-infected HEK-293T cells, since they express repellents of neurite outgrowth. We show that HEK-293T cells secrete factors that inhibit neurite outgrowth, while infection with HSV-2 strains MS and 333 reduces this repelling phenotype, increasing neurite numbers. The HSV-2-mediated restoration of neurite outgrowth required the activity of NGF. In the absence of infection, however, NGF did not overcome the repulsion mediated by HEK-293T cells. We previously showed that recombinant, soluble glycoprotein G of HSV-2 (rSgG2) binds and enhances NGF activity, increasing neurite outgrowth. However, the effect of gG2 during infection has not been investigated. Therefore, we addressed whether gG2 contributes to overcoming neurite outgrowth repulsion. To do so, we generated viruses lacking gG2 expression and complemented them by exogenous expression of gG2. Overall, our results suggest that HSV-2 infection of nonneuronal cells reduces their repelling effect on neurite outgrowth in an NGF-dependent manner. gG2 contributed to this phenotype, but it was not the only factor. The enhanced neurite outgrowth may facilitate HSV-2 spread from epithelial cells into neurons expressing NGF receptors and increase HSV-2-mediated pathogenesis.

**IMPORTANCE** Herpes simplex virus 2 (HSV-2) is a prevalent human pathogen that establishes lifelong latency in neurons of the peripheral nervous system. Colonization of neurons is required for HSV-2 persistence and pathogenesis. The viral and cellular factors required for efficient infection of neurons are not fully understood. We show here that nonneuronal cells repel neurite outgrowth of sensory neurons, while HSV-2 infection overcomes this inhibition and, rather, stimulates neurite outgrowth. HSV-2 glycoprotein G and nerve growth factor contribute to this phenotype, which may attract neurites to sites of infection and facilitate virus spread to neurons. Understanding the mechanisms that modulate neurite outgrowth and facilitate HSV-2 infection of neurons might foster the development of therapeutics to reduce HSV-2 colonization of the nervous system and provide insights on neurite outgrowth and regeneration.

## Abstract (precis)

Herpes simplex virus 2 (HSV-2) is a prevalent human pathogen that establishes lifelong latency in neurons of the peripheral nervous system. Colonization of neurons is required for HSV-2 persistence and pathogenesis. The viral and cellular factors required for efficient infection of neurons are not fully understood. We show here that nonneuronal cells repel neurite outgrowth of sensory neurons, while HSV-2 infection overcomes this inhibition and, rather, stimulates neurite outgrowth. HSV-2 glycoprotein G and nerve growth factor contribute to this phenotype, which may attract neurites to sites of infection and facilitate virus spread to neurons. Understanding the mechanisms that modulate neurite outgrowth and facilitate HSV-2 infection of neurons might foster the development of therapeutics to reduce HSV-2 colonization of the nervous system and provide insights on neurite outgrowth and regeneration.

## INTRODUCTION

Herpes simplex virus 1 (HSV-1) and HSV-2 establish lifelong latency in sensory neurons of the trigeminal and dorsal root ganglia (DRG) as well as in autonomic neurons (1–7). They cause a variety of diseases, including cold sores, genital herpes, keratitis, and encephalitis. To infect sensory neurons, HSV enters neurites in the mucosa or the skin and reaches the soma by retrograde axonal transport (8, 9). Neurites are dynamic structures that grow or retract when exposed to attractive or repulsive cues, respectively. These include axon guidance molecules (AGM) and neurotrophic factors. There are more than 50 AGM, including members of the semaphorins, ephrins, etrins, and slits, and 40 receptors (10). Depending on the cellular context, the same AGM may be attractive or repulsive (11). There are also many neurotrophic factors, including those of the neurotrophin and glial cell line-derived neurotrophic factor (GDNF) families. Cells in the mucosa and skin express attractive and repulsive cues that act on their receptors present in neurons (12). Repulsive cues, such as semaphorin 3A and class A ephrin, repel neurite outgrowth, while attractive ones, such as interleukin 17c (IL-17c) and nerve growth factor (NGF), increase neurite outgrowth and survival (13).

Despite the relevant role of AGM and neurotrophic factors in neurite outgrowth and neuronal survival, their modulation by HSV-2 infection is not well understood. Peng and colleagues reported that upon reactivation in humans, HSV-2 induces IL-17c expression in keratinocytes of the genital tract, leading to subsequent neurite outgrowth (14). The authors suggested that the higher level of IL-17c increased neuronal survival during recurrent HSV-2 reactivation (14). We showed previously that recombinant, soluble glycoprotein G from both HSV-1 and HSV-2 (rSgG1 and rSgG2, respectively) bind NGF but that only rSgG2 increases NGF-induced neurite outgrowth (15). Moreover, rSgG1 and rSgG2 bind chemokines and enhance their potency, increasing leukocyte migration (16). Chemokines and neurotrophic factors are involved in the interplay between the immune and nervous systems, with chemokines antagonizing axonal repulsion and with IL-17c, NGF, and others regulating the innate immune response and inducing inflammation (17–20). gG is the most divergent glycoprotein between HSV-1 and HSV-2. Both gG1 and gG2 are type I transmembrane proteins, which are incorporated into the viral envelope (21–24). However, proteolytic processing of the mature gG2 (MgG2) leads to the secretion of its N-terminal domain (22, 23). The chemokine and NGF binding activities are located in the gG1 ectodomain and the secreted N-terminal domain of gG2, termed here SgG2 (15, 16). SgG2 is also detected in purified virions, either due to inefficient processing or due to the binding of SgG2 to the envelope through glycosaminoglycans, and is functionally active, since it binds chemokines and enhances their activity (21, 25).

Here, we addressed whether HSV-2 infection reduces the repelling effect of nonneuronal cells on neurite outgrowth. We also addressed the role of NGF and gG2 in this process. We established an *ex vivo* neurite outgrowth system that models the repelling effect of nonneuronal HEK-293T cells on mouse DRG neurons. This system permits a mechanistic analysis not amenable *in vivo*. Mouse DRGs serve as a valid model to study several aspects of HSV neurobiology *ex vivo* (26–32). Our results showed that infection of HEK-293T cells with either of two HSV-2 strains reduced the repelling effect of uninfected cells, facilitating neurite outgrowth, in an NGF-dependent manner. The use of the recombinant HSV-2 MS strain lacking gG2 expression and complementation experiments showed that gG2 participated in this activity but that it was not the sole factor. It is noteworthy that the effect of gG2 on neurite outgrowth was less relevant during infection with the HSV-2 333 strain than during infection with the HSV-2 MS strain. The reduced repulsion of neurite outgrowth during HSV-2 infection of nonneuronal cells may facilitate the colonization of the nervous system and impact pathogenesis.

## RESULTS

### Factors secreted into the medium of nonneuronal cells repel NGF-dependent neurite outgrowth of sensory neurons.

**FIG 1:**
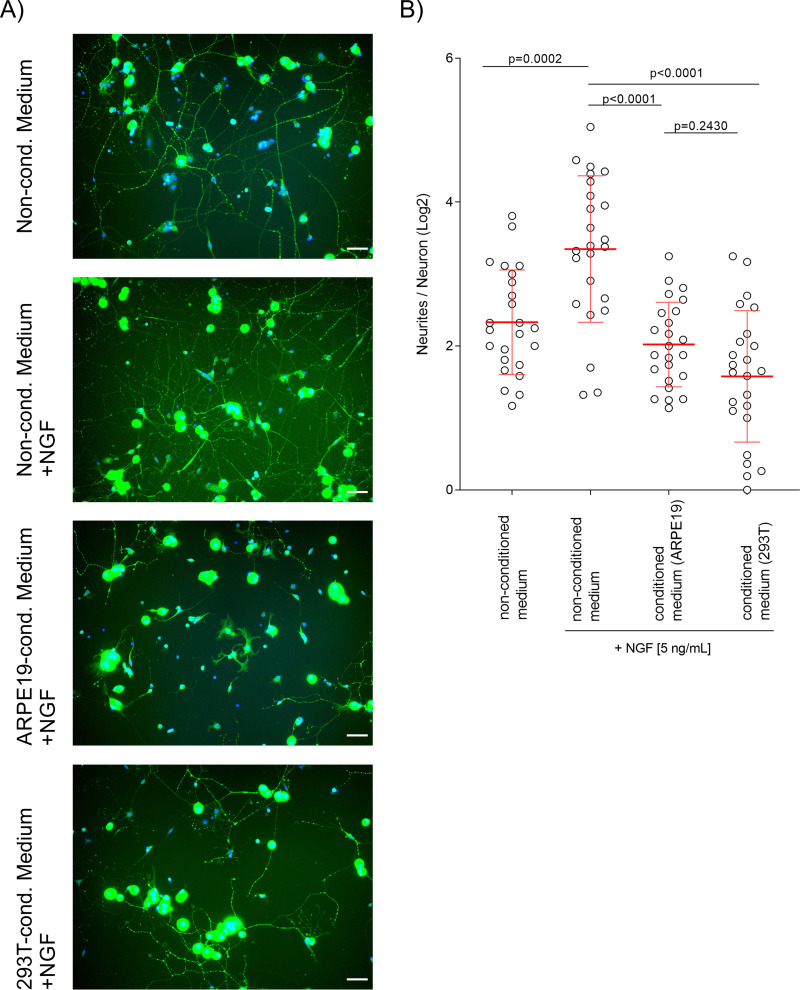
HEK-293T and ARPE-19 cells secrete inhibitors of neurite growth. (A) Representative images of primary DRG-derived mouse neurons exposed to different media with or without NGF. Neurons were labeled with an anti-tubulin β-III antibody (Tuj1; green), and the DNA was labeled with DAPI (blue). Bars, 50 μm. (B) Graph showing the number of neurites per neuron (log_2_ transformed) following incubation of cells with conditioned medium from HEK-293T or ARPE-19 cells or nonconditioned medium with or without NGF. The results are from one representative experiment out of two independent ones. The means for the population and the standard deviation (error bars) are depicted. *P* values were calculated using ANOVA with Browne-Forsythe and Welch corrections with Dunnett’s T3 multiple-comparison posttest for indicated groups.

To address the impact of HSV-2 infection of nonneuronal cells on neurite outgrowth, we first characterized the repelling effect of factors secreted by two cell lines, HEK-293T and ARPE-19, into the culture medium. We incubated primary neurons obtained from mouse DRG with NGF plus conditioned medium from HEK-293T or ARPE-19 cells collected at 72 h postseeding. As a control, we used nonconditioned cell culture medium with or without NGF. After 24 h of incubation, we labeled neurites with antibodies against the neuronal marker tubulin β-III (Fig. 1A). We counted the neurites and neurons in regions of interest (ROI). Figure 1B shows the number of neurites per neuron in an equal number of ROI per condition after log_2_ transformation. Neurons cultured for 24 h in nonconditioned medium without NGF had about 5 neurites (mean, 5.03 neurites; limits of the 95% confidence interval [CI], 4.07 to 6.22 neurites) (Fig. 1B). NGF induced neurite outgrowth (mean, 10.18 neurites; 95% CI, 7.56 to 13.72 neurites). Conditioned supernatants from ARPE-19 cells (mean, 4.06 neurites; 95% CI, 3.42 to 4.82 neurites) or HEK-293T cells (mean, 2.99 neurites; 95% CI, 2.28 to 3.90 neurites) abolished the stimulating effect of NGF on neurite outgrowth. These results indicate that NGF induces neurite outgrowth of mouse DRG neurons and that factors secreted by both ARPE-19 and HEK-293T cells prevent this induction, results comparable to previously obtained results with sympathetic neurons (15).

### Generation of a recombinant reporter HSV-2.

**FIG 2:**
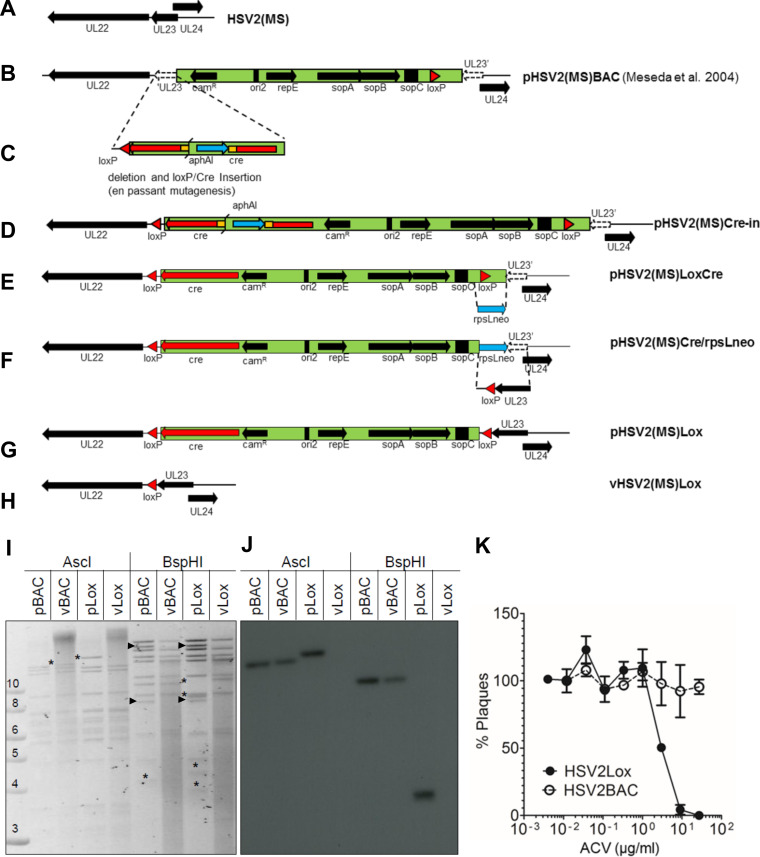
Construction of the self-excisable, TK-positive BAC pHSV2(MS)Lox. (A to H) Schematic representation of the DNA constructs and HSV-2 intermediates during the modification of pHSV2(MS)BAC to pHSV2(MS)Lox, as described in Materials and Methods. cam^R^, chloramphenicol resistance cassette; ori2, repE, sopA, sopB, and sopC, genes and loci required for BAC replication in E. coli; cre, eukaryotic Cre recombinase expression cassette, driven by the CMV promoter; *loxP*, Cre recombinase target sites; *aphAI*, kanamycin resistance cassette; *rpsLneo*, kanamycin resistance cassette-streptomycin resistance cassette fusion. (I) Agarose gel showing the results of restriction analysis using AscI and BspHI of BAC DNA from pHSV2(MS)BAC (pBAC) and pHSV2(MS)Lox (pLox) and the corresponding viral DNA isolated from eukaryotic cells transfected with the respective BACs (vBAC and vLox). The size markers on the left are in kilobase pairs. Changes in the restriction pattern resulting from BAC mutagenesis are indicated by asterisks. (J) Southern blot analysis. DNA restriction fragments from the agarose gel in panel I were transferred to a nylon membrane and probed with a ^32^P-labeled PCR amplicon of the BAC-encoded chloramphenicol resistance cassette. (K) Determination of the acyclovir sensitivity of HSV2(MS)BAC and HSV2(MS)Lox. The relative number of plaques (considering the number of plaques with the smallest amount of ACV to be 100%) from triplicate titrations is plotted against the indicated concentrations of acyclovir (mean ± standard deviation).

To monitor HSV-2 infection, we generated HSV-2 reporter viruses using the MS strain containing the bacterial artificial chromosome (BAC) [HSV2(MS)BAC], provided by Meseda and colleagues (33). A BAC cassette in HSV2(MS)BAC had been inserted into the *UL23* gene, to disrupt thymidine kinase (TK) expression (Fig. 2) (33). We repaired the *UL23* gene and introduced a self-excisable Cre recombinase-expressing cassette into pHSV2(MS)BAC by *en passant* mutagenesis (34) to generate HSV2(MS)Lox (Fig. 2A to H). Enzymatic restriction analyses and Southern blot analysis confirmed the complete excision of the BAC cassette after HSV-2 reconstitution in eukaryotic cells (Fig. 2I and J). We confirmed the repair of the *UL23* gene and the expression of functional TK with an acyclovir (ACV)-based plaque reduction assay in U2OS cells (Fig. 2K) and determined a half-maximal inhibitory concentration of ACV of 2.9 μg/ml (12.9 μM), within the expected range (35). ACV at 24 μg/ml completely arrested the replication of HSV2(MS)Lox in U2OS cells, whereas that of HSV2(MS)BAC, lacking a functional TK, was unaffected. Thus, in contrast to HSV2(MS)BAC, HSV2(MS)Lox expressed a functional TK.

**FIG 3:**
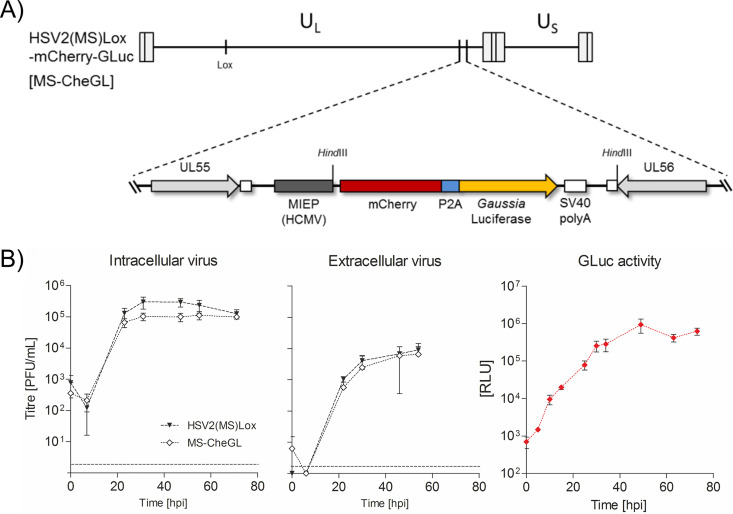
Generation and characterization of the HSV-2 MS reporter strain. (A) Schematic representation of the HSV-2 MS-CheGL genome with its unique long (U_L_) and unique short (U_S_) regions, the internal and terminal repeats, and the inserted Lox site. The enlarged region shows the mCherry-P2A-GLuc reporter cassette between ORFs *UL55* and *UL56*. SV40, simian virus 40. (B) Growth curves of the parental HSV2(MS)BAC and the MS-CheGL reporter strain (MS-CheGL) in Vero cells. The intracellular and extracellular infectious HSV-2 isolates collected at different times postinfection were titrated on Vero cells. The *Gaussia* luciferase activity in the supernatant of infected cells was also measured. Data are plotted as the average from three biologically independent replicate experiments, with error bars showing standard deviations. Abbreviations: hpi, hour postinfection; RLU, relative light units. The dashed lines near the bottom of each graph indicate the limits of detection. Data points below the limits of detection represent the results for cultures devoid of plaques.

We used HSV2(MS)Lox to generate the reporter HSV2(MS)Lox-_pHCMV_mCheGLuc (named MS-CheGL for short) expressing a bicistronic mRNA under the control of the major IE promoter (MIEP) of human cytomegalovirus (HCMV) and encoding mCherry and *Gaussia* luciferase (GL) separated by a picornavirus 2A (P2A) site (36) (Fig. 3A). We introduced the reporter cassette in the intergenic locus between the open reading frames (ORFs) of *UL55* and *UL56*, a locus that does not affect HSV-1 replication (37). We sequenced the virus obtained upon reconstitution in mammalian cells (the sequences have been uploaded into GenBank and may be found under accession no. MH796783). The replication of the MS-CheGL virus was similar to that of its parental HSV2(MS)Lox virus in HEK-293T cells (Fig. 3B), indicating that the insertion of the reporter cassette did not affect HSV-2 replication, as reported before for HSV-1 (37). We also detected secreted *Gaussia* luciferase (GLuc) in the supernatant. The level of GLuc activity was similar to the overall kinetics of virus replication (Fig. 3B).

### HSV-2 infection reduces the repelling effect of HEK-293T cells.

**FIG 4:**
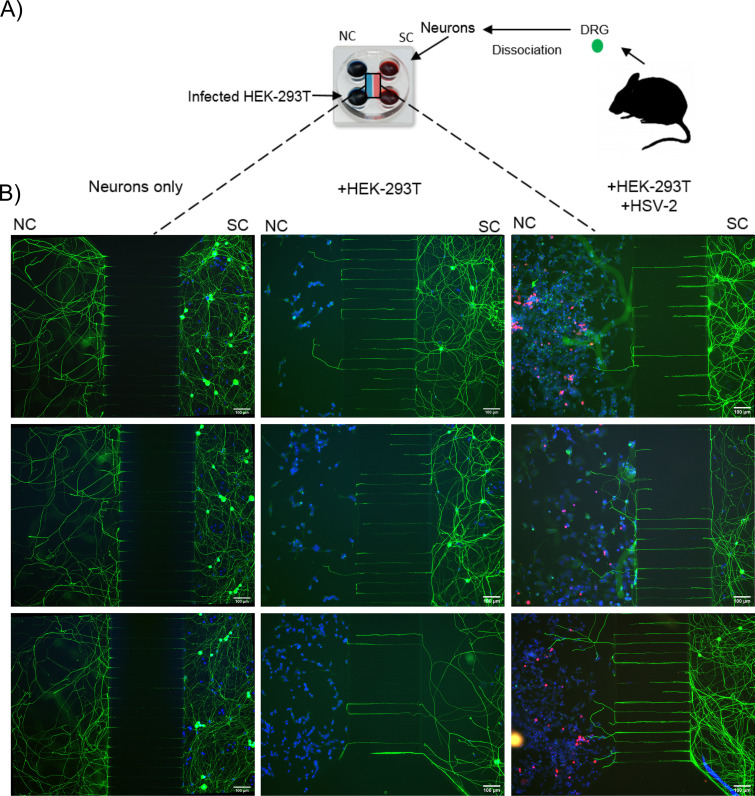
Experimental setup to measure the effect of HSV-2 infection of nonneuronal cells on neurite outgrowth. (A) Schematic representation of the MFC containing HEK-293T cells and mouse DRG cells. We seeded dissociated mouse DRG neurons in the somal compartment (SC). After 24 h, we added HSV-2-infected (MOI, 0.1) or mock-treated HEK-293T cells to the neurite compartment (NC) in the presence of aphidicolin. Seventy-two hours later the cells were fixed and stained. We acquired images of the channel on the NC side (indicated by the boxed area) and quantified neurite outgrowth. (B) Representative images showing neurite outgrowth from the SCs through the microgrooves to the NCs. The left side of each image shows the neurites and HEK-293T cells in the NCs, whereas the right part contains the neurons seeded in the SC. Neurons were labeled with an anti-tubulin β-III (Tuj1 antibody; green), DNA was detected with DAPI (blue), and infected cells were identified by mCherry expression (magenta). The number and length of neurites entering the NCs from the microgrooves were quantified with the Simple Neurite Tracer plug-in of ImageJ software.

Based on the results shown in Fig. 1, we decided to address the impact of HSV-2 infection on the repelling effect of HEK-293T cells. We chose HEK-293T cells over ARPE-19 cells because they are easier to transfect and express repellents of neurite outgrowth, such as semaphorin 3A and class A ephrin (38, 39). We seeded mouse DRG neurons in microfluidic chambers (MFC) to separate the cell bodies and neurites (Fig. 4A). These devices have two compartments connected by microgrooves that allow the passage of neurites but not neuronal cell bodies and have been widely used to study alphaherpesvirus neurobiology (32, 40–42). We placed dissociated DRG neurons from mice aged between 33 and 38 days in the somal compartment (SC) and seeded mock- or HSV-2-infected HEK-293T cells into the neurite compartment (NC). NGF was added to both the NC and the SC. We seeded a mix of 20% infected cells (mCherry positive) and 80% noninfected cells into the NC to mimic the situation at the initial phase of infection, when most of the cells are not infected. We seeded the same number of dissociated DRG cells and HEK-293T cells under all conditions in every experiment (5 × 10^4^ and 2 × 10^5^, respectively). As a control, we cultured dissociated neurons without HEK-293T cells. The HEK-293T cells were infected for 24 h and added to the NC 1 day after neuronal seeding. At this time, the neurites had already entered the NC from the SC through the microgrooves. We added the HEK-293T cells with aphidicolin to inhibit any further mitosis, as this would lead to high cell confluence, precluding the observation of neurites. Aphidicolin also inhibits HSV replication (43–45). Aphidicolin reduced HSV-2 titers by about 1 log unit after 24 h in HEK-293T cultures compared to that for the controls (data not shown).

**FIG 5:**
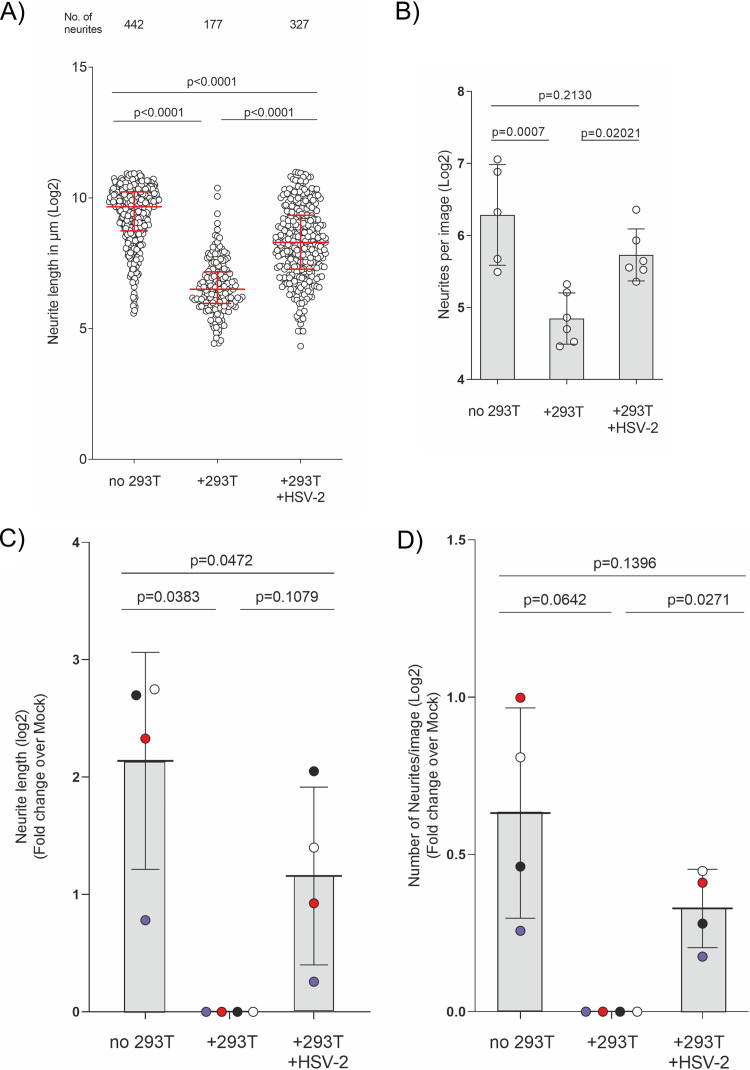
HSV-2 infection of nonneuronal cells reduces their repulsion of neurite outgrowth. (A) Quantification of neurite length and number in one representative experiment out of four (log_2_ transformed). Each data point represents one neurite. The total number of neurites is shown for every condition in the top row. The data were not normally distributed; therefore, the medians for the populations (red) and the interquartile range (error bars) are depicted. *P* values were calculated using the Kruskal-Wallis test with Dunn’s multiple-comparison posttest for the indicated groups. (B) Number of neurites detected per image (log_2_ transformed). Data points represent the number of neurites counted per image for the experiment whose results are shown in panel A. The means for the population and the standard deviation (error bars) are depicted. *P* values were calculated using ANOVA with Sidak’s multiple-comparison posttest for the indicated groups. (C, D) Normalized neurite length (C) and number of neurites (D) from four independent experiments. Data points belonging to the same experiment have the same color. The means for the population and the standard deviation (error bars) are depicted. *P* values were calculated using a random-block ANOVA with Geisser-Greenhouse corrections with Tukey’s multiple-comparison posttest for all groups.

After 3 days of coculturing HEK-293T cells and neurons, we detected the neurons with an antibody to tubulin β-III, the nuclei with DAPI (4′,6-diamidino-2-phenylindole), and infected cells by HSV-2-mediated mCherry expression. There were many long neurites in the absence of HEK-293T cells (Fig. 4B, left), whereas the presence of mock-treated HEK-293T cells reduced the number and length of neurites (Fig. 4B, middle). This suggests that the HEK-293T cells expressed repellents of neurite outgrowth, as shown before (Fig. 1) (38, 39, 46). Infection of HEK-293T cells with HSV-2 (MS-CheGL) partially overcame the repulsion, with more and longer neurites growing toward the HEK-293T cells (Fig. 4B; compare the middle and right panels). To measure neurite outgrowth, we traced their length with the Simple Neurite Tracer tool in ImageJ software (47). We measured the neurite length and number from the edge of the microgrooves into the NC (Fig. 5 A and B). Quantification of the neurite length showed shorter neurites in the presence of HEK-293T cells (median, 90.88 μm; limits of the interquartile range [IQR], 62.5 to 144.4 μm) than in neurons alone (median, 811 μm; limits of IQR, 487.9 to 1.202 μm) (Fig. 5A). This negative effect on neurite length was reduced when the NC contained infected HEK-293T cells (median, 314.7 μm; limits of IQR, 154.3 to 647.5 μm). The same trend was observed when comparing the total number of neurites (Fig. 5A, top line) and the number of neurites per image (Fig. 5B). There were 442 neurites in total and 77.98 neurites/image (mean; 95% CI, 42.72 to 142.32 neurites/image) in the NC without HEK-293T cells, while the presence of HEK-293T cells resulted in 177 neurites and 28.74 neurites/image (mean; 95% CI, 22.19 to 37.25 neurites/image). The presence of infected cells in the NC increased the total number of neurites to 327 and the number of neurites per image to 53.01 (mean; 95% CI, 40.76 to 68.93 neurites/image). Infection of HEK-293T cells with HSV-2 reduced their repulsion over that for mock-infected cells in four independent experiments with primary neurons, resulting in longer neurites (mean length, 2.23-fold increase; 95% CI, 0.97- to 5.14-fold increase) and more neurites (mean number, 1.3-fold increase; 95% CI, 1.1- to 1.44-fold increase) (Fig. 5C and D). The increase in neurite number was more robust than the increase in neurite length. These results show that the presence of NGF without HSV-2 infection did not revert the inhibitory phenotype and that HSV-2 infection of HEK-293T cells partially inhibited their repulsion on neurite outgrowth.

### gG2 expression contributes to inhibit the repulsion of HEK-293T cells during infection.

**FIG 6:**
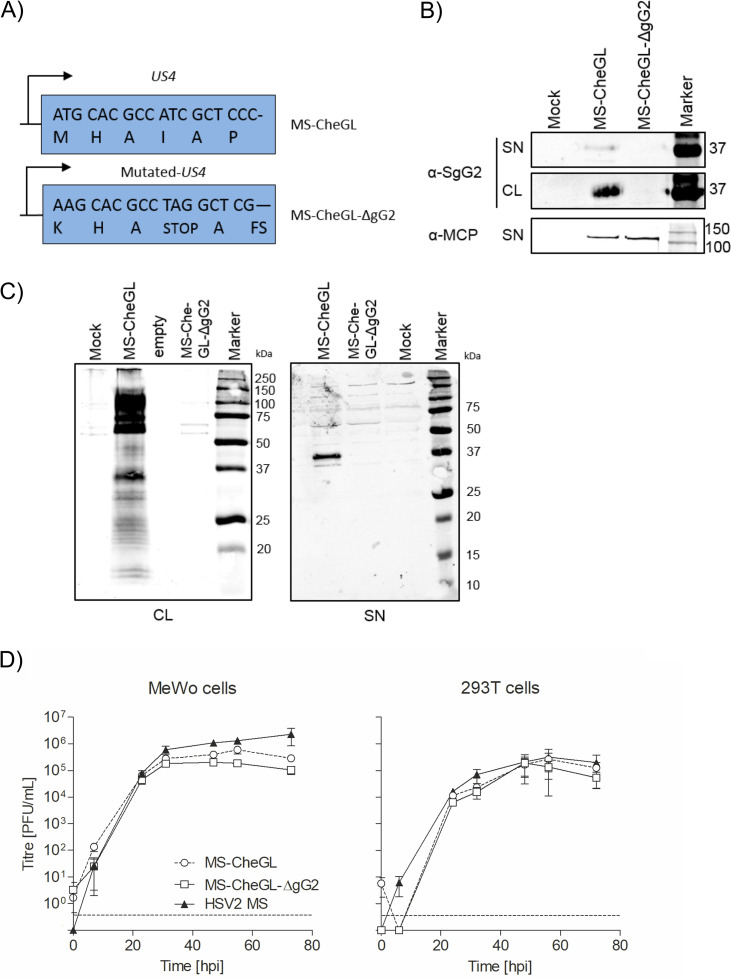
Construction and characterization of a gG2-deficient HSV-2 strain, MS-CheGL-ΔgG2. (A) Mutations introduced in the *US4* gene of HSV-2 MS-CheGL to generate HSV-2 MS-CheGL-ΔgG2 that lacks expression of gG2. The 3′ 18 nucleotides and corresponding residues are shown. FS, frameshift. (B, C) Detection of gG2 and the major capsid protein (MCP) by immunoblotting in the supernatant and cell lysate (SN and CL, respectively) of MS-CheGL- and MS-CheGL-ΔgG2-infected Vero cells. (C) Western blots showing a lack of gG2 expression and the absence of truncated gG2 proteins in the cell lysates and supernatant of MS-CheGL-ΔgG2-infected cells. The antibodies used target the gG2 ectodomain (CL, anti-MgG2; left) and the secreted N-terminal domain (SN, anti-SgG2; right). In panel B, the numbers to the right of the gel are molecular weights (in kilodaltons). (D) Multistep growth curves of parental HSV-2 MS strain, MS-CheGL, and MS-CheGL-ΔgG2 viruses in MeWo (left) and HEK-293T (right) cells. Cell lysates of infected cells (MOI, 0.01) were collected at the indicated time points, and a plaque assay was performed in Vero cells. Data points represent the mean for three cultures per time point. Error bars represent standard deviations. Abbreviation: hpi, hour postinfection. The dashed lines near the bottom of each graph indicate the limit of detection. Data points below the limit of detection represent the results for cultures without plaques.

Repellents secreted from HEK-293T cells into the conditioned medium, exogenous NGF, and HSV-2 infection modulated neurite outgrowth in our model. Purified rSgG2 increases NGF-induced neurite outgrowth (15). Whether the gG2 expressed during infection has a similar function was not known. To address the role of gG2 in neurite outgrowth during infection, we generated an HSV-2 mutant lacking gG2 expression [HSV2(MS)Lox-Che-GLuc-ΔgG2, or MS-CheGL-ΔgG2 for short]. We mutated the *US4* start codon and introduced a stop codon and a frameshift to prevent gG2 expression (Fig. 6A). This strategy reduces the possibility of gG2 expression, even if there were a restoration of the ATG codon. We sequenced MS-CheGL-ΔgG2 upon reconstitution in mammalian cells (GenBank accession no. MH796784). There were no undesired mutations in the recombinant virus. We could not detect gG2 in lysates or supernatants from MS-CheGL-ΔgG2-infected cells, while the levels of the major capsid protein (MCP) were unaffected (Fig. 6B). We also addressed whether translation could start at methionine 286, producing a shorter form of gG2 of 418 amino acids. This would correspond to a protein with a molecular weight of approximately 45 kDa. We did not detect any part or precursor form of the gG2 protein using an antibody targeting residues 290 to 321 or the gG2 ectodomain, which would have been included in an N-terminally truncated gG2 (Fig. 6C). The lack of gG2 expression did not affect replication in MeWo and HEK-293T cells (Fig. 6D).

**FIG 7:**
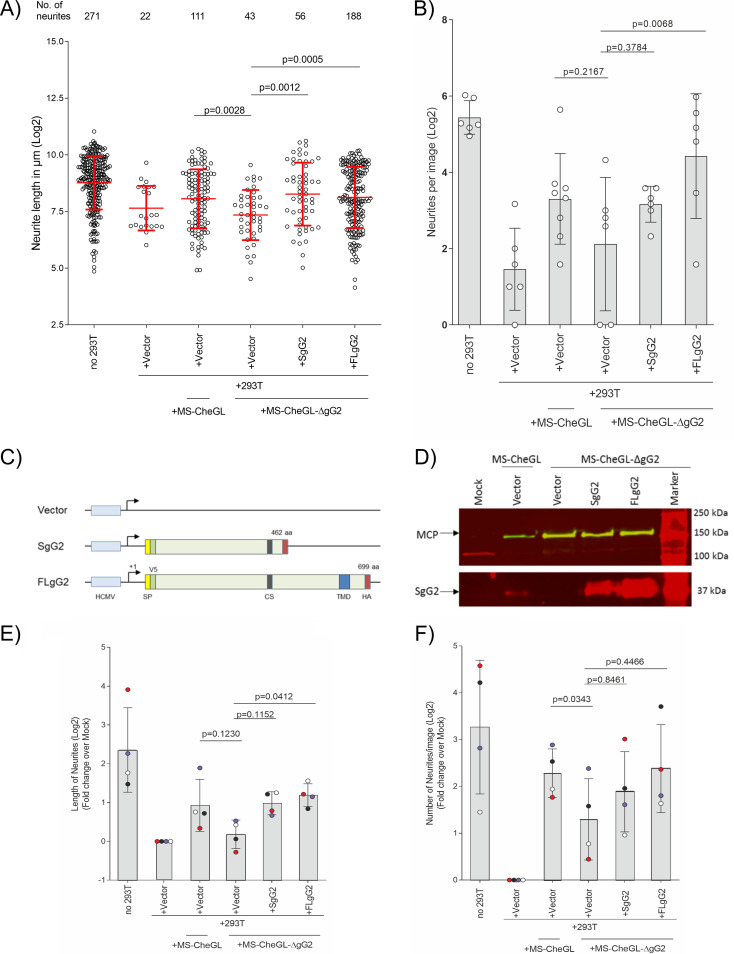
Glycoprotein G from HSV-2 contributes to reduce the neurite repulsion of nonneuronal cells during HSV-2 infection. Dissociated DRG neurons were seeded as described in the legend to Fig. 4. HEK-293T cells were either mock transfected or transfected with plasmids expressing rSgG2 or recombinant full-length gG2 (rFLgG2) and subsequently infected (MOI, 0.1) at 4 h posttransfection. At 72 h after seeding HEK-293T cells into the SC, cells were fixed and labeled for tubulin β-III and neurite outgrowth was quantified. The means for the population and the standard deviation (error bars) are depicted in all graphs. (A) Quantification of the neurite length and number from one representative experiment out of four (log_2_ transformed). Each data point represents one neurite. The total number of neurites is given for every condition in the top row. *P* values were calculated using ANOVA with Browne-Forsythe and Welch corrections with Dunnett’s T3 multiple-comparison posttest for the indicated groups. (B) The number of neurites per image from one representative experiment out of four is shown. *P* values were calculated using ANOVA with Sidak’s multiple-comparison posttest for the indicated groups. (C) Schematic representation of the transfected plasmids. The Signaling peptide (SP), V5 and hemagglutinin (HA) tags, the cleavage site of the protein (CS), and the transmembrane domain (TMD) of the C-terminal domain are indicated by colored boxes. (D) Western blots showing the expression of MCP (green) and SgG2 (red) in infected and cotransfected HEK-293T cells (the plasmids used are indicated above each lane). (E, F) Graphs showing the log_2_-transformed normalized neurite length (E) and number of neurites (F) measured in four independent experiments performed as described in the legend to panel A. Data sets were normalized to the average data for the mock infection condition (+293T +Vector) of each experiment before plotting. Data points belonging to the same experiment have the same color. *P* values were calculated using a random-block ANOVA with the Geisser-Greenhouse correction and Sidak’s multiple-comparison posttest for the indicated groups.

Next, we characterized the impact of HEK-293T cells infected with parental or gG2-deficient HSV-2 on neurite outgrowth (Fig. 7). As before (Fig. 5), mock-infected HEK-293T cells inhibited neurite outgrowth, while infection with the parental virus partially reverted the phenotype, inducing the formation of longer and more neurites (Fig. 7A and B). The neurites were longer in the absence (mean, 436.25 μm; 95% CI, 395.63 to 481.37 μm) than in the presence (mean, 200.44 μm; 95% CI, 148.26 to 270.97 μm) of HEK-293T cells. Infection of HEK-293T cells with HSV-2 resulted in neurites longer than those in the noninfected control (mean, 267.24 μm; 95% CI, 225.50 to 316.71 μm). The MS-CheGL-ΔgG2 virus was less efficient than the MS-CheGL virus in reducing the repulsion by HEK-293T cells (mean, 162.56 μm; 95% CI, 128.44 to 205.93 μm). This suggested that the observed effects were not simply caused by HSV-2-infection but were at least partially due to gG2 expression. To test whether gG2 contributed to counteract the repressive effect on neurite outgrowth, we complemented MS-CheGL-ΔgG2 in *trans* with gG2-expressing constructs. We transfected HEK-293T cells with an empty control or plasmids expressing SgG2 (pSgG2) or full-length gG2 (FLgG2) (pFLgG2) prior to infection with the MS-CheGL-ΔgG2 virus (Fig. 7C). We plated transfected and infected HEK-293T cells in the NC of MFC 1 day after seeding dissociated DRG cells and quantified neurite outgrowth 72 h later. Complementation led to a partial rescue of the MS-CheGL-ΔgG2 phenotype (for pSgG2, a mean of 308.26 μm [95% CI, 238.20 to 398.93 μm]; for pFLgG2, a mean of 278.59 μm [95% CI, 243.54 to 318.68 μm]; for the vector, a mean of 162.56 μm [95% CI, 128.44 to 205.93 μm]) (Fig. 7A). Similar effects were also detectable in the number of neurites (Fig. 7A, top line). There were 271 and 22 neurites in the absence or presence of HEK-293T cells, respectively. Infection of HEK-293T cells with MS-CheGL resulted in 111 neurites, while 43 neurites were observed upon infection with MS-CheGL-ΔgG2. The complementation in *trans* with SgG2 or FLgG2 increased the neurite numbers to 56 and 188, respectively (Fig. 7A). Similarly, there were 43.38 neurites/image (mean; 95% CI, 31.47 to 59.80 neurites/image) and 2.75 neurites/image (mean; 95% CI, 1.26 to 6.01 neurites/image) in the absence and presence of HEK-293T cells, respectively. Infection with MS-CheGL increased the neurite density to 9.87 neurites/image (mean; 95% CI, 4.96 to 19.66 neurites/image), while infection with MS-CheGL-ΔgG2 led to only 4.34 neurites/image (mean; 95% CI, 1.22 to 15.51 neurites/image). Complementation with SgG2 or FLgG2 increased the number of neurites per image above the level achieved with MS-CheGL-ΔgG2 alone (for pSgG2, a mean of 8.96 neurites/image [95% CI, 6.36 to 12.64 neurites/image]; for pFLgG2, a mean of 21.50 neurites/image [95% CI, 6.54 to 70.67 neurites/image]) (Fig. 7B).

Transfection with pFLgG2 complemented MS-CheGL-ΔgG2 more efficiently than transfection with pSgG2. The expression of gG2 and MCP was assessed by immunoblotting in HEK-293T cell cultures (Fig. 7D). Transfection of pFLgG2 resulted in more SgG2 expression than transfection of pSgG2, despite equal molarities of the transfected plasmids (Fig. 7D) (48). This difference in protein levels may have been responsible for the higher neurite outgrowth observed when pFLgG2 was transfected. However, we cannot exclude the possibility that other domains of gG2, such as the ectodomain, the transmembrane region, or the cytoplasmic tail, also contribute to neurite outgrowth.

We compared the results from four independent experiments after normalizing the average length or the average number of neurites per image to those for the mock-infected sample for each data set (Fig. 7E and F). We obtained results similar to those presented in Fig. 7A, with MS-CheGL-ΔgG2 infection leading to shorter neurites (mean length, 1.14-fold increase; 95% CI, 0.75- to 1.70-fold increase) than infection with MS-CheGL (mean length, 1.90-fold increase; 95% CI, 0.91- to 3.99-fold increase) in all four experiments (Fig. 7E and F). Transfection with pSgG2 partially complemented the MS-CheGL-ΔgG2 phenotype (mean length, 1.98-fold increase; 95% CI, 1.42- to 2.74-fold increase). Complementation was more efficient with pFLgG2 (mean length, 2.28-fold increase; 95% CI, 1.65- to 3.16-fold increase) than with pSgG2. When comparing the number of neurites, we observed the same trend. In all four independent experiments, there were fewer neurites when cells were infected with MS-CheGL-ΔgG2 (mean number, 2.45-fold increase; 95% CI, 0.94- to 6.39-fold increase) than when they were infected with MS-CheGL (mean number, 4.86-fold increase; 95% CI, 2.74- to 8.62-fold increase). Transfection with pSgG2 or pFLgG2 did not significantly increase the neurite number (for pSgG2, the mean was a 2.45-fold increase [95% CI, 1.44- to 9.51-fold increase]; for pFLgG2, the mean was a 5.20-fold increase [95% CI, 1.84- to 14.64-fold increase]). Overall, our results showed that the MS-CheGL-ΔgG2 virus was less efficient than its parental MS-CheGL virus in overcoming the repulsion of HEK-293T cells. Complementation with gG2 in *trans* partially reverted this phenotype, indicating that HSV-2 gG contributed to reduce the repulsion of neurite outgrowth, but it was not the only factor involved.

### Infection with HSV-2 strain 333 increases neurite outgrowth in an NGF-dependent manner.

**FIG 8:**
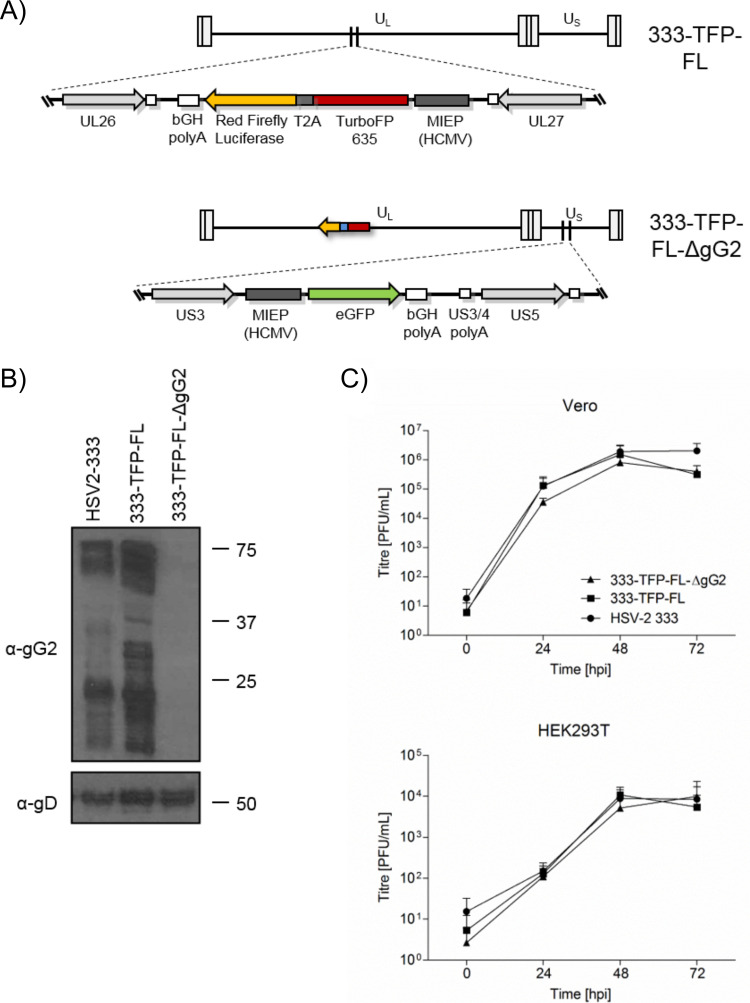
Construction and characterization of HSV-2 333-TFP-FL-ΔgG2 lacking gG2 expression. (A) Schematic representation of the genomes of HSV-2 333-TFP-FL and 333-TFP-FL-ΔgG2 with their unique long (U_L_) and unique short (U_S_) regions and the internal and terminal repeats. The enlarged region in 333-TFP-FL shows the presence of the TurboFP635-T2A-RedFirefly reporter cassette between the *UL26* and *UL27* ORFs. The enlarged region in 333-TFP-FL-ΔgG2 shows the replacement of the *US4* gene with an EGFP expression cassette. (B) Immunoblots showing gG2 and gD (top and bottom blots, respectively) expression in cell lysates of cells infected with wild-type HSV-2 strain 333, 333-TFP-FL, and 333-TFP-FL-ΔgG2. The numbers to the right of the gel are molecular weights (in kilodaltons). (C) Multistep growth curves of wild-type HSV-2 strain 333, 333-TFP-FL, and 333-TFP-FL-ΔgG2 viruses in Vero and HEK-293T cells (top and bottom graphs, respectively). Cell lysates of infected cells (MOI, 0.01) were collected at the indicated time points, and a plaque assay was performed in Vero cells. Data represent the averages from three independent experiments, with error bars showing the standard deviations. Abbreviation: hpi, hour postinfection.

To determine whether the HSV-2-enhanced neurite outgrowth was strain specific, we constructed another HSV-2 mutant lacking gG2 expression using CRISPR/Cas9 (Fig. 8). First, we generated a reporter virus, HSV2(333)_pHCMV_TurboFP635-FRFL (333-TFP-FL, in short), by inserting TurboFP635 (TFP) and red firefly luciferase (FL) under the control of the MIEP promoter of HCMV between the *UL26* and *UL27* genes, a locus previously shown to permit the insertion of foreign DNA without affecting virus replication (49). We replaced the *US4* locus, coding for gG2, with a cassette expressing enhanced green fluorescent protein (EGFP), generating HSV2(333)_pHCMV_TurboFP635-FRFL-FLuc-ΔgG2, or 333-TFP-FL-ΔgG2 in short (Fig. 8A). These viruses were plaque purified and sequenced (the sequences have been uploaded to the European Nucleotide Archive and may be found under accession no. ERS3367584 [https://www.ebi.ac.uk/ena/browser/view/ERS3367584] and ERS3367585 [https://www.ebi.ac.uk/ena/browser/view/ERS3367585]). There were no undesired mutations in these viruses. Infection with 333-TFP-FL-ΔgG2 led to a lack of gG2 protein expression. However, there were similar levels of envelope glycoprotein gD upon infection with 333-TFP-FL-ΔgG2 or the parental virus, 333-TFP-FL (Fig. 8B). Both 333-TFP-FL and 333-TFP-FL-ΔgG2 replicated with kinetics similar to those for their parental wild-type HSV-2 333 strain (Fig. 8C).

The effect of HSV-2 333 infection on neurite length was minor. There were longer neurites in the absence than in the presence of HEK-293T cells (means, 1,136.199 μm [95% CI, 1,089.92 to 1,184.45 μm] and 713.61 μm [95% CI, 653.93 to 778.21 μm], respectively). Infection with 333-TFP-FL increased the mean length to 826.00 μm (95% CI, 774.43 to 881.00 μm). Infection with 333-TFP-FL-ΔgG2 resulted in a mean length similar to that achieved by infection with the parental strain (mean, 798.42 μm; 95% CI, 735.71 to 866.47 μm). This result differed from that obtained with the MS strain, in which the MS-CheGL-ΔgG2 virus induced shorter neurites than the MS-CheGL virus. To determine the functional relevance of NGF, we added an anti-NGF neutralizing antibody. We used the antibody and did not simply remove NGF from the culture because DRG cells also express and release NGF. Treatment of the culture with this NGF antibody prior to and during incubation with infected HEK-293T cells prevented the slight increase in neurite length induced by 333-TFP-FL (mean, 759.55 μm; 95% CI, 701.84 to 822.57 μm).

**FIG 9:**
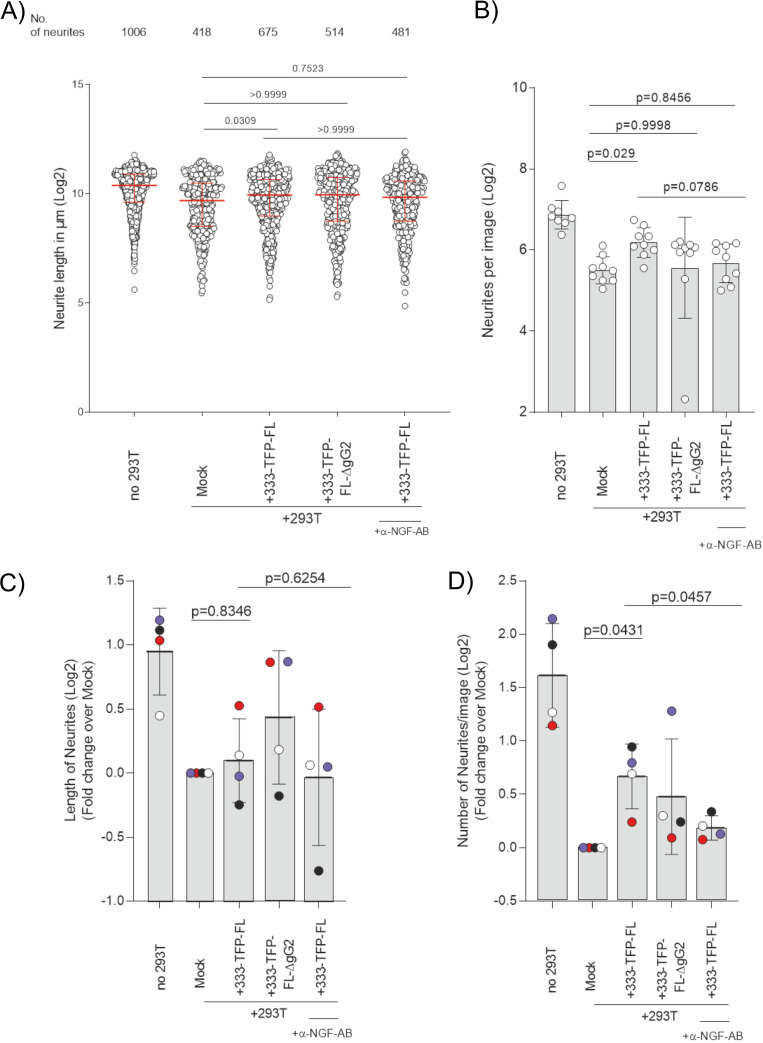
Glycoprotein G2 from HSV-2 strain 333 and NGF contribute to overcome the repulsion of neurite outgrowth, increasing the neurite number. Dissociated mouse DRG neurons were seeded in the somal compartment (SC) and mock treated or HSV-2 infected (333-TFP-FL and 333-TFP-FL-ΔgG2 strains; MOI, 0.1) HEK-293T cells were added to the neurite compartment (NC) 24 h later. Under one condition, a neutralizing anti-NGF antibody was added together with the 333-TFP-FL-infected cells. Forty-eight hours later the cells were fixed and neurite outgrowth was quantified after staining the neurites with anti-tubulin β-III antibody. The means for the population and the standard deviation (error bars) are depicted in all graphs. (A) The graph shows the log_2_-transformed length of the neurites under each experimental condition. Each data point represents one neurite. The total number of neurites is given for every condition in the top row. One representative experiment out of four independent ones is shown. *P* values were calculated using ANOVA with Browne-Forsythe and Welch corrections with Dunnett’s posttest for multiple testing to compare the indicated group means. (B) Log_2_-transformed number of neurites per image from the experiment whose results are shown in panel A. *P* values were calculated using ANOVA with the Browne-Forsythe and Welch correction with Dunnett’s posttest for multiple testing to compare the indicated group means. (C, D) Summary of the data from four independent experiments as described in the legends to panels A and B, plotted after normalization to the results for mock-infected cells (with HEK-293T cells only [+293T]) and log_2_ transformation. Data points belonging to the same experiment have the same color. *P* values were calculated using a random-block ANOVA with the Geisser-Greenhouse correction and Sidak’s multiple-comparison posttest for indicated groups. AB, antibody.

Infection of HEK-293T cells with 333-TFP-FL increased the number of neurites in the presence of NGF compared to the number for mock-infected cells (Fig. 9A and B). This effect was similar to that of the HSV-2 MS strain (Fig. 5D). Infection with the 333-TFP-FL virus increased the neurite number from 418 (with HEK-293T cells only) to 675, while infection with the 333-TFP-FL-ΔgG2 virus did not (514 neurites). Addition of the NGF-neutralizing antibody reduced the number of neurites to 481 (Fig. 9A, top). The same trend was visible when the numbers of neurites per image were compared (Fig. 9B). There were 117.13 neurites/image (mean; 95% CI, 95.67 to 143.41 neurites/image) in the absence of HEK-293T cells, while their presence reduced this number to 45.32 neurites/image (mean; 95% CI, 37.98 to 54.08 neurites/image). Infection of HEK-293T cells with the 333-TFP-FL virus increased the number of neurites per image to 72.91 (mean; 95% CI, 59.96 to 88.65 neurites/image). In contrast, infection with the 333-TFP-FL-ΔgG2 virus did not increase the number of neurites per image (mean, 47.27 neurites/image; 95% CI, 24.35 to 91.84 neurites/image) compared to that achieved in the presence of HEK-293T cells. Furthermore, addition of the NGF-neutralizing antibody to the cells infected with the 333-TFP-FL virus reduced the number of neurites per image to 51.02 (mean; 95% CI, 39.62 to 65.75 neurites/image), similar to the level observed for mock-treated HEK-293T cells. We compared the results from four independent experiments after normalizing the average length and number of neurites per image to those for the mock-infected sample for each data set (Fig. 9C and D). We did not observe a significant effect on neurite length (Fig. 9C). The effects on the number of neurites were reproducible and followed the same trend that was seen upon infection with HSV-2 MS. In all four experiments, infection with the 333-TFP-FL virus increased the number of neurites, whereas in three out of four experiments, the 333-TFP-FLΔgG2 virus failed to do so (Fig. 9D). We also observed that the neutralization of NGF prevented the increase in neurite number by 333-TFP-FL infection in all experiments. These results indicate that gG2 of both HSV-2 MS and 333 increased the neurite number during infection in an NGF-dependent manner. They also suggest that there might be strain-specific differences in the effect sizes between HSV-2 MS and 333.

## DISCUSSION

The first, main objective of this project was to determine whether HSV-2 modulates the neurite outgrowth of sensory neurons during infection of nonneuronal cells. Second, we wanted to characterize the role of NGF and gG2 in this process. Our results show that NGF increased the neurite outgrowth of DRG neurons and that repellents secreted by HEK-293T and ARPE-19 cells inhibited this growth. Infection with the HSV-2 MS or 333 strain counteracted the repelling effect of HEK-293T cells in an NGF-dependent manner, having a stronger impact on neurite number than on neurite length. Infection with HSV-2 MS lacking gG2 expression resulted in fewer and shorter neurites than infection with the parental virus. Complementation experiments showed that gG2 contributed to the inhibition of the repulsive effect on neurite outgrowth but that it was not the sole factor involved. The neurites were longer and more numerous in the presence of gG2 and NGF, suggesting that these proteins cooperated in counteracting the HEK-293T cell-mediated neurite repulsion. Interestingly, infection with HSV-2 strain MS increased both neurite length and neurite number, whereas strain 333 increased the number of neurites but not their length. Moreover, gG2 did not contribute to neurite outgrowth during infection with HSV-2 strain 333, pointing to potential strain-specific differences. Addition of NGF-neutralizing antibodies reduced HSV-2-induced neurite outgrowth, demonstrating the contribution of NGF to this process.

Complementation with FLgG2 in *trans* confirmed that gG2 counteracted the HEK-293T cell-mediated repulsion of neurite outgrowth and that other potential mutations in the HSV-2 genome were not responsible for this phenotype. Supporting this, we did not find significant differences between the genomes of MS-CheGL and MS-CheGL-ΔgG2 or 333-TFP-FL and 333-TFP-FL-ΔgG2 by next-generation sequencing. FLgG2 is proteolytically cleaved, leading to the secretion of SgG2 and retention of the remaining domains of the gG2 type I transmembrane protein in the cells (21–23, 48). The fact that FLgG2 complements CheGL-ΔgG2 more efficiently than SgG2 is interesting and requires further investigation. This may be due to higher protein levels upon transfection of pFLgG2. However, the ectodomain, transmembrane region, or cytosolic tail of gG2 may also actively contribute to overcoming the repulsion of neurite outgrowth by modifying the expression or activity of AGMs and neurotrophic factors. The ectodomain of gG2 is 298 amino acids long and may interact with other cellular proteins involved in neurite outgrowth. There are many AGMs and neurotrophic factors that could be modulated by FLgG2. It will be interesting to determine whether FLgG2 modulates their expression or activity.

Our results also suggest that gG2 is not the only factor overcoming the repelling effect of nonneuronal cells during infection. HEK-293T cells express repellents, such as semaphorin 3A and class A ephrins (38, 39, 46). The possibility that HSV-2 infection may also modulate the expression or activity of attractive or repulsive cues affecting neurite outgrowth, in addition to the gG-mediated modulation reported here, requires further investigation. One could also postulate the presence of alternative indirect or direct effects of other viral factors on the expression of AGMs. For example, HSV modifies gene expression and protein translation by several mechanisms, including disruption of transcription termination (50, 51). Moreover, the virus host shutoff (VHS) protein, encoded by *UL41*, induces both transcription and translation shutoff (52, 53). A potential direct effect of a viral gene could be mediated by HSV-2-infected cell protein 0, since this protein induces IL-17c expression during infection of human keratinocytes (14).

The different impact of HSV-2 MS or 333 infection on neurite outgrowth may have been due to strain-specific effects on the expression or activity of IL-17c or AGMs. This seems more likely than differences in gG2 from these two strains, as their amino acid identity is 98% (only 9 out of 698 residues are different). Moreover, we showed previously that gG2 from HSV-2 333 enhances NGF-induced neurite outgrowth (15). However, we cannot formally exclude the possibility that these differences at the amino acid level are relevant to enhance NGF function during infection. The fact that NGF activity was required to inhibit the repelling effect of HEK-293T cells on the number of neurites, even during HSV-2 infection, suggests that the observed effect is not only due to a nonspecific effect on gene expression in the infected nonneuronal cells.

Our results have several implications for HSV-2 persistence and pathogenesis. Following cell entry at the neurites, HSV-2 establishes lifelong latency in peripheral neurons. Upon reactivation, newly generated virions exit the neurons via the neurites and cause genital herpes, characterized by itch, burn, and pain (54, 55). Proinflammatory cytokines expressed during infection by epithelial cells and infiltrating leukocytes may damage neurites and induce neuronal activity and death. However, some of these cytokines—e.g., tumor necrosis factor alpha, IL-1β, and IL-6—also induce the expression of NGF by epithelial cells, fibroblasts, and neurons (56–59). Neurotrophic factors send survival signals to the neuronal cell body, reducing neurotoxicity. In this context, HSV-2 reactivation induces the expression of IL-17c in keratinocytes, increasing neurite density and, potentially, neuronal survival in the human genital tract (14). NGF promotes neurite outgrowth, neuronal survival, and neuronal differentiation upon binding to tropomyosin receptor kinase A (TrkA) (60, 61). NGF and other neurotrophins also bind to the p75 neurotrophin receptor (p75NTR), a member of the tumor necrosis factor receptor superfamily (62, 63). Our previous results indicated that rSgG2 acts mainly on the NGF-TrkA axis (15). Activation of the NGF-TrkA axis increases the survival of responsive neurons, but it may also have pathological consequences, such as the induction of pain (60).

Sensory ganglia contain a diverse population of neurons with different expression and functional profiles (64). TrkA is often coexpressed with the calcitonin gene-related peptide (CGRP) in peptidergic neurons that express the transient receptor potential vanilloid 1 (TRPV1), involved in the sensation of painful stimuli (65, 66). NGF induces pain through enhancing the expression and activation of TRPV1 and sodium channels, and injection of rSgG2 in the mouse footpad increases the sensation of thermal pain through TrkA and TRPV1 activation (30, 67–69). Interestingly, HSV-2 replicates more efficiently in neurons that express CGRP and TrkA (70, 71), and its replication is restricted in neurons lacking TrkA and expressing the GDNF receptor, probably leading to the establishment of latency (72, 73). Moreover, NGF and GDNF signaling are required to maintain HSV-1 and HSV-2 latency, respectively (74–76). Therefore, enhanced NGF activity and the subsequent increase in neurite outgrowth may facilitate HSV-2 spread from infected epithelial cells to sensory neurons and promote the survival of neurons expressing TrkA. This could favor initial HSV-2 spread and may contribute to the induction of acute, local pain.

Overall, we show that HSV-2 infection reduced the repulsion of neurites and increased their growth in an NGF-dependent manner. The contribution of gG2 was strain specific, indicating that this was not the sole factor involved. Whether other alphaherpesviruses modulate the activities of neurotrophic factors or AGM to increase neurite outgrowth is currently unknown. We propose that HSV-1 and HSV-2 have developed several strategies to modulate neurite outgrowth and neuronal survival. The discovery and characterization of such mechanisms of neuronal modulation may provide novel therapeutic targets for intervention to either limit the infection of neurons by HSV or reduce virus-induced tissue damage.

## MATERIALS AND METHODS

### Ethics statement.

No experiments with living animals were performed in this study. Animals were sacrificed for scientific purposes according to regulation Tierschutzgesetz §4, in strict accordance with the German regulations of the Society for Laboratory Animal Science (GV-SOLAS), the European Health Law of the Federation of Laboratory Animal Science Association (FELASA), and the German Animal Welfare Law. Mice were bred and hosted at the Animal Facility of Hannover Medical School (Hannover, Germany) following the highest standard of animal care under specific-pathogen-free conditions. Routine microbiological monitoring according to FELASA recommendations (77) did not reveal any evidence of infection with common murine pathogens, except for Staphylococcus aureus and Helicobacter hepaticus. The total number of sacrificed animals was reported with animal welfare application number 2018/191, according to §4 of the German Animal Welfare Law at the LAVES (Niedersaechsisches Landesamt fuer Verbraucherschutz und Lebensmittelsicherheit, Oldenburg, Germany).

### Mice.

BALB/cJ-Ztm mice (ZTL, MHH Hannover) 35 days old (±3 days) were used in this study. The animals were housed and bred in the onsite Animal Facility of Hannover Medical School.

### Cell lines and viruses.

Human embryonic kidney epithelial (HEK-293T) cells (ATCC-CRL-3216), human epithelial (ARPE-19) cells (ATCC-CRL-2302), and human melanoma (MeWo) cells (ATCC-HTB-65) were cultured in Dulbecco modified Eagle medium (DMEM) supplemented with 8% fetal calf serum (FCS), penicillin-streptomycin (Pen-Strep), and 5 mM l-glutamine. Human osteosarcoma (U2OS) cells (ATCC-HTB-96) were cultured in DMEM supplemented with 10% FCS, 2 mM l-glutamine, 1 mM sodium pyruvate, and 3.75 mg/ml sodium bicarbonate. Cercopithecus aethiops kidney epithelial Vero cells (ATCC-CCL-81) were cultured in Eagle minimal essential medium supplemented with 8% FCS. All mammalian cells were grown at 37°C in a CO_2_ buffered cell incubator.

The HSV2-BAC MS strain [termed HSV2(MS)BAC in this report] was kindly provided by Jerry Weir (Food and Drug Administration, Bethesda, MD, USA) and has been previously described (33). The generation and characterization of HSV2(MS)Lox, HSV2(MS)Lox-_pHCMV_mCheLuc (termed MS-CheGL in this report), and HSV2(MS)Lox-_pHCMV_mCheLuc-ΔgG2 (termed MS-CheGL-ΔgG2 in this report) are described below. HSV-2 strain 333 was kindly provided by Helena Browne (University of Cambridge, Cambridge, UK). The generation of recombinant viruses using this strain is explained below.

### Generation of recombinant viruses. (i) Construction of the self-excisable, TK-positive BAC pHSV2(MS)Lox.

The BAC replication origin was inserted into the pHSV2(MS) genome by homologous recombination; thereby, the thymidine kinase (TK)-encoding gene (*UL23*) was disrupted (33) (Fig. 2A and B). We flanked the nonviral sequences with *loxP* sites and inserted a eukaryotic Cre recombinase expression cassette to remove the BAC replication origin from the viral backbone upon transfection of eukaryotic cells (78, 79). To this end, we performed *en passant* BAC mutagenesis (34) using a DNA fragment amplified from the plasmid pEP-Cre-in using primers TAT TGC CGT CAT CGC CGG GAG GCC TTC CGT TCG GGC TTC CGT GTT TGA AAT AAC TTC GTA TAA TGT ATG C and CCA AGC TAT TTA GGT GAC ACT ATA GAA TAC TCA AGC TTG ACC CCC CAG GCT ATA GGG CGA ATT GGA GCT C (Fig. 2C). The template pEP-Cre-in was generated by inserting an I-SceI site, a kanamycin resistance cassette (*aphAI*), and a 50-bp duplication into pUC18LC (78). This DNA fragment was amplified from pEP-Kan-S (80) with TTA AGG ATC CAC AAG GAT GAC GAT AAG and TTA AGG ATC CGA AAA GAA AAC GTT GAT GCC GGT GAA CGT GCA AAA CAG GCT CTA GCG TTC CAG GGT AAT GCC AGT GTT AC and inserted via BamHI. After *en passant* mutagenesis, the remaining 3′ end of the disrupted *UL23* had been removed (Fig. 2E). In the resulting BAC, pHSV2(MS)LoxCre, the wrongly oriented *loxP* site at the 3′ end of the BAC origin insertion was replaced by bacteriophage Red recombination with a rpsLneo cassette amplified from pRpsL-neo (Gene Bridges, Dresden, Germany) with primers TCT CTG TTT TTG TCC GTG GAA TGA ACA ATG GAA GTC CGA GCT CAT CGC TAG CCT GGT GAT GAT GGC GGG ATC G and CTA TCG CCT CCC TGC TGT GCT ACC CGG CCG CGC GGT ACC TCA TGG GAA GCT CAG AAG AAC TCG TCA AGA AGG (Fig. 2F). This cassette was replaced under streptomycin counterselection with a DNA fragment containing a correctly oriented *loxP* site and the complete *UL23* ORF, which was amplified from HSV-2 DNA with the primers TCT CTG TTT TTG TCC GTG GAA TGA ACA ATG GAA GTC CGA GCT CAT CGC TAA TAA CTT CGT ATA ATG TAT GCT ATA CGA AGT TAT CTA AAC TCC CCC CAC CTC GCG and ATG GCT TCT CAC GCC GGC CAA C (Fig. 2G). In the resulting BAC, pHSV2(MS)Lox, the *UL23* ORF encoding TK was restored, and the nonviral sequences were flanked with *loxP* sites for Cre-mediated excision after the transfection of eukaryotic cells (Fig. 2H). All oligonucleotides were synthesized by Sigma.

### (ii) Construction of the pHSV2(MS)Lox-mCherry-GLuc BAC (MS-CheGL) and *US4* (gG2)-deficient mutant pHSV2(MS)Lox-mCherry-GLuc-ΔgG2 BAC (MS-CheGL-ΔgG2).

To construct the MS-CheGL reporter virus, an mCherry-GLuc cassette expressing a kanamycin resistance cassette and the two reporter genes separated by a picornavirus 2A (P2A) peptide was used. The P2A peptide prevents normal peptide bond formation without inhibiting translation (81). The cassette was amplified from plasmid Pm157-hMIEP-mCherry-GLuc using primers Reportercassette-homolbox-fwd1 (GAG AAC CAA CCA AAA CAG ACG CGG TGT GAG TTT GTG GGT TTC GAG ATC CTA GTT ATT AAT AGT AAT CAA TTA C) and Reportercassette-homolbox-rev1 (CAC ATG CTG GTT CGT CGC GTG GTA TTT ACC GGG TTC CTA TGC GTT CAT GTA TGC GAC TAG TCT AC) carrying 40- bp overhangs homologous to the target sequences in the pHSV2(MS)Lox BAC between ORFs *UL55* and *UL56* (Fig. 3), producing pHSV2(MS)Lox-mCherry-GLuc, named MS-CheGL in this study. The plasmid carrying the reporter cassette is based on Pm157-mMIEP-mCherry-GLuc (36) and was constructed by replacing the murine cytomegalovirus (MCMV) MIEP (mMIEP) with the HCMV MIEP. The primer sequences binding to plasmid Pm157-mMIEP-mCherry-GLuc are underlined. The cassette was inserted into the BAC using *en passant* BAC mutagenesis, as described above for pHSV2(MS)Lox BAC.

To mutate the *US4* ORF in the HSV-2 MS genome, a kanamycin resistance cassette flanked by overhangs containing the stop codon and frameshift mutations was produced by PCR. Primers with overhangs containing the 40-bp sequences targeting *US4*, the stop codon and frameshift mutations, and a short repeat for the seamless removal of the kanamycin resistance gene cassette were used. The kanamycin resistance gene cassette was amplified from plasmid pEP-KanS2 (80) using primers MutgG2-fwd1 (GCG GCC CTC GGG CTT TGG TGT TTT TGG CAC CTT GCC GCC **CAA GCA CGC CTA GGC TCG** AGG TTG CTT CTT CTT TTT GTT CTT TCT AGG GAT AAC AGG GTA ATC GAT TT) and MutgG2-rev1 (CGC GTG TCC CCG GAA GAC CAG AAA GAA CAA AAA GAA GAA **GCA ACC TCG AGC CTA GGC GTG CTT** GGG CGG CAA GGT GCC AAA AAG CCA GTG TTA CAA CCA ATT AAC C), and the resulting cassette was incorporated into the HSV2-(MS)LoxBAC by *en passant* mutagenesis (bold letters indicate changes in the *US4* sequence). After confirming the successful mutagenesis of *US4*, this BAC was also tagged with mCherry-GLuc using the same approach described above, producing HSV2(MS)Lox-mCherry-GLuc-ΔgG2, named MS-CheGL-ΔgG2 in this study. The successful incorporation of the BAC cassette and the successful mutagenesis of the *US4* ORF were checked by PCR, enzymatic restriction analysis, and next-generation sequencing of the viral genome (with an Illumina MiSeq system) (see below).

### (iii) Generation of strain 333 recombinant viruses.

All sequences refer to the HSV-2 strain 333 complete genome (GenBank accession no. LS480640 [82]). The detailed procedure to generate recombinant viruses with the CRISPR/Cas9 system will be described elsewhere (A. D. López-Muñoz, A. Rastrojo, and A. Alcami, unpublished data). To generate the donor vector, pF_UL26-27(2)_CMV-Red-bGH(Rv), to recombine with the HSV-2 genome, the UL26-27 region (nucleotides 52,250 to 53,249) was cloned into a modified version of pcDNA3.1/Zeo(−) (Invitrogen, Life Technologies, Carlsbad, CA, USA) by In-Fusion cloning (Clontech Laboratories, Mountain View, CA, USA) to make pF_UL26-27(2). This modified pcDNA3.1/Zeo(−) was generated by high-fidelity (HF) PCR amplification, excluding the HCMV enhancer/promoter-bGH region, by the use of the following primers: pcDNA1.0_Fw (CTT CTG AGG CGG AAA GAA CCA) and pcDNA1.0_Rv (AAC GCG TAT ATC TGG CCC G). The humanized far-red fluorescent protein TurboFP635 (scientific name, Katushka) was amplified from pTurboFP635-N (Evrogen, Moscow, Russia). The 2A peptide from the Thosea asigna virus capsid protein (T2A) was amplified from pSpCas9(BB)-2A-Puro V2.0 (Addgene plasmid 62988). These fragments were then cloned into pCMV-RedFireflyLuc (Thermo Fisher Scientific, MA, USA) by In-Fusion cloning between the T7 promoter and red firefly luciferase (RedFireflyLuc) to construct pCMV_TurboFP635-T2A-RedFireflyLuc_bGH. The CMV_TurboFP635-T2A-RedFireflyLuc_bGH cassette (Red cassette) was amplified from this previous plasmid and cloned in the reverse orientation into pF_UL26-27(2) by In-Fusion cloning, generating pF_UL26-27(2)_CMV-Red-bGH(Rv). The Red cassette was inserted in pF_UL26-27(2) at the reference positions between nucleotides 52,749 and 52,750.

To construct the donor vector pF_US4(2)_CMV-eGFP-bGH to replace the *US4* gene, its genomic region (nucleotides 137,411 to 140,507) was cloned into the modified pcDNA3.1/Zeo(−) without the HCMV enhancer/promoter-bGH region by In-Fusion cloning, generating pF_US4(2). This vector was linearized by HF PCR amplification, excluding the *US4* ORF. The CMV-eGFP-bGH cassette (Green cassette) was then amplified from pEGFP-N3 (Clontech Laboratories, Mountain View, CA, USA) and cloned, by In-Fusion cloning, into this previously linearized vector.

The plasmids used to target the *UL26-27* site of insertion in the HSV-2 333 genome were constructed as follows: pCas9(gRNA3_2) was constructed by annealing two complementary oligonucleotides (CAC CGA AGG CCC GGA AGA CTA CCA C and AAA CGT GGT AGT CTT CCG GGC CTT C), and pCas9(gRNA7_2) was constructed with the CAC CGC CGG AAG ACT ACC ACG GGG A and AAA CTC CCC GTG GTA GTC TTC CGG C oligonucleotides. For Cas9 targeting of the *US4* site of insertion, pCas9(gRNA10_US4-2) was constructed using the CAC CGA TGG CCA CAC AAG CCG CAA and AAA CTT GCG GCT TGT GTG GCC ATC oligonucleotides, and pCas9(gRNA25_US4-2) was constructed with the annealed CAC CGC GAT TCG CCT ACG TCC GCT C and AAA CGA GCG GAC GTA GGC GAA TCG C oligonucleotides. The resulting double-stranded DNA fragment, for each case, was ligated in the BbsI site of pSpCas9(BB)-2A-Puro (version 2.0) (83). These oligonucleotides, used as guide RNA (gRNA) by Cas9 nuclease, were designed by using Protospacer Workbench software, as previously described (84). To generate HSV2(333)_pHCMV_TurboFP635-FRFL (in short, 333-TFP-FL), HEK-293T cells were transfected with the donor plasmid pF_UL26-27(2)_CMV-Red-bGH and two puromycin-resistant plasmids [pCas9(gRNA3_UL26-27_2) and pCas9(gRNA7_UL26-27_2)] encoding Cas9 and two different gRNAs for insertion into the intergenic locus between *UL26* and *UL27*. After puromycin selection, cells were infected with wild-type strain HSV-2 333. At 2 days postinfection (dpi), cells were harvested, subjected to three freeze-thaw cycles, and 10-fold serially diluted to infect Vero cells. The 333-TFP-FL virus was identified by fluorescence microscopy, plaque purified, and sequenced. To generate the HSV2(333)_pHCMV_TurboFP635-FRFL-ΔgG2 virus (in short, 333-TFP-FL-ΔgG2), HEK-293T cells were transfected with the donor plasmid pF_US4(2)_CMV-eGFP-bGH and two puromycin-resistant Cas9-gRNA plasmids targeting the *US4* locus [pCas9(gRNA10_US4_2) and pCas9(gRNA25_US4_2)]. To obtain the 333-TFP-FL-ΔgG2 virus, cells were infected with the 333-TFP-FL virus after puromycin selection, and virus identification, purification, and sequencing were performed as described above.

### (iv) Production of virus stocks.

The virus stocks for this study were produced from the supernatant and lysed infected Vero cells. In short twenty 15-cm plates with Vero cells were infected at a multiplicity of infection (MOI) of 0.01 and incubated for at least 72 h until a full cytopathic effect was visible. Then, the cells were scraped off and pelleted into 50-ml Falcon tubes. The supernatant was kept on ice, while the cell pellets were disrupted by 3 freeze-thaw cycles. The cell debris was then pelleted in a Heraeus tabletop centrifuge (860 × *g*, 5 min, 4°C), and the supernatant was then combined with the supernatant from the first round of centrifugation. Virus was then pelleted using a Beckman L8-70 ultracentrifuge (type 19 rotor, 12,000 rpm, 4°C, 90 min), and the pellet was resuspended in 1 ml of cell culture medium.

### Analytical preparation of BAC DNA.

BAC DNA for analytical purposes was isolated from transformed Escherichia coli GS 1783 cells (80) overnight culture using a NucleoBond XBAC kit from Macherey-Nagel (Düren, Germany).

### Restriction analysis of BAC DNA.

To analyze the restriction fragments of BAC DNA and viral DNA from HSV2(MS)Lox and HSV2(MS)BAC, we used AscI and BspHI (New England Biolabs). The DNA fragments were separated on 0.6% agarose in 0.5× TBE (45 mM Tris-borate, 1 mM EDTA, pH 8.0) for 16 h at 2.8 V/cm. Restriction analysis of HSV2(MS)Lox-mCherry-GLuc (MS-CheLuc) and HSV2(MS)Lox-mCherry-GLuc-ΔgG2 (MS-CheLuc-ΔgG2) was carried out using HindIII (New England Biolabs) and separating the fragments on a 0.8% agarose gel in 1× TAE (40 mM Tris, 20 mM acetic acid, 1 mM EDTA, pH 8.0) for 16 to 20 h (Fig. 2).

### Southern blot analysis.

DNA was digested and separated by electrophoresis. The gels were blotted onto nylon membranes (GE Healthcare, Little Chalfont, UK) and hybridized with an [alpha-^32^P]dCTP-labeled probe directed against the chloramphenicol resistance gene encoded in the BAC replication origin (78). The radioactive probe was generated using a random primed DNA labeling kit (Roche, Mannheim, Germany). The hybridization signals were visualized by autoradiography.

### Acyclovir plaque reduction assay.

A total of 5 × 10^5^ U2OS cells/well were seeded into 6-well plates and infected with HSV-2 at an MOI of 0.001 PFU/cell for 1 h at room temperature. Afterwards, the virus inoculum was removed and 2 ml culture medium containing 25 μg/ml immunoglobulin G (IgG) and ACV was added. At 2 dpi, the viral plaques were counted to determine the viral titer. Experiments were conducted in triplicate. ACV dilutions with concentrations ranging from 0 to 27 μg/ml were prepared in culture medium. The 50% inhibitory concentration value was determined with the calculated virus titer at the given ACV concentration. HSV-neutralizing IgG (25 μg/ml; Sigma-Aldrich Chemie GmbH) was added to the culture medium to prevent reinfection of the cells with progeny virions secreted into the medium.

### Next-generation sequencing. (i) NGS of BAC-derived viruses (MS strain).

Sequencing of BAC-derived strain MS viruses was performed at the Institute of Virology, Hannover Medical School. The integrity of all recombinant HSV-2 isolates was verified by whole-genome deep sequencing. Briefly, purified BAC DNA and isolated viral genomic DNA from virus reconstituted in mammalian cells were sheared by sonication. To avoid bias by overamplification, library preparation was performed using a Kapa real-time library preparation kit (Kapa Biosystems, Wilmington, MA, USA). Libraries were sequenced on a MiSeq system (Illumina), using the reagent kit (version 3) to generate 2 × 300-base paired-end reads. The reads were mapped to HSV-2 HG52 as a reference strain (GenBank accession no. NC_001798). Genetic variants (variants with single-nucleotide polymorphisms and insertions/deletions) were identified by using the low-frequency variant detector function in CLC Genomics Workbench software. Variants were considered valid under the following conservative criteria: an overall read depth of ≥50, an average base call quality of ≥20, a forward/reverse read balance of 0.3 to 0.5, and a variant frequency of ≥2% (i.e., the relative frequency of a variant at a particular position). Variants detected within homopolymer regions were excluded. Sequence reads were *de novo* assembled, and the resulting contigs were oriented against an HSV-2 reference strain lacking the terminal repeat long (TRL) and terminal repeat short (TRS) regions. *De novo*-assembled sequences contain small gaps in repeat regions and are marked as a stretch of nucleotides (85). The sequences are listed as a partial genome in GenBank (MH796783 and MH796784) .

### (ii) NGS of strain 333 recombinant viruses.

Libraries were sequenced on a MiSeq system (Illumina) using a reagent kit (version 3) to generate 2 × 250-base paired-end reads at MicrobesNG (University of Birmingham, Birmingham, UK). Reads from HSV-2 strain 333-derived viruses were mapped to the HSV-2 333 reference genome (GenBank accession no. LS480640). Genetic variants were identified by using VarScan (version 2.4.3) (86) under the following criteria: a minimal coverage of ≥20, a variant frequency of ≥2%, and a strand filter of ≥90.

### Replication kinetics in eukaryotic cells.

Eighty percent confluent HEK-293T and MeWo cells in 48-well plates were infected with 100 μl virus inoculum (MS strain; MOI, 0.01) for 1 h at 37°C. The inoculum was removed, and the cells were washed once with phosphate-buffered saline (PBS). At the time points indicated in the corresponding figure legends, the supernatant and cells from 3 wells per virus were harvested separately. Supernatants or cell lysates (subjected to 3 freeze-thaw cycles) were then titrated (10× dilution series, 100-μl inoculum) in parallel on Vero cells in triplicate. After 3 days, plaques and infected mCherry-positive foci were counted with a microscope.

For replication kinetics with the HSV-2 333 wild-type strain and derived viruses, confluent Vero and HEK-293T cells grown in 12-well plates were infected with 400 μl virus inoculum at an MOI of 0.01 for the multistep growth curves. Then, the cells were overlaid with 1 ml fresh DMEM containing 2% fetal bovine serum. At the time points specified above, supernatant or cells (subjected to 3 freeze-thaw cycles) from 2 wells per virus were harvested and titrated (10× dilution series) on Vero cells in duplicate. The cells were stained with crystal violet, and the plaques were manually counted at 2 dpi.

### *Gaussia* luciferase assay.

To measure the GLuc activity in infected cell cultures, we used a microplate luminometer (Orion II; Berthold) with an injector system. Fifty microliters of culture supernatant was plated into a 96-well opaque white plate (Nunc) and, by using the injector, was mixed with 50 μl 1-μg/ml native coelenterazine (a 1-mg/ml stock in methanol [Sigma] diluted in PBS). Enzymatic activity was measured in the well mode with the following sequence: injection (50 μl), with no shaking, delay for 0 s, measurement at 10 s. The values were analyzed with GraphPad Prism (version 5.0) software after using the remove baseline function.

### Western blotting.

Cells were lysed in radioimmunoprecipitation assay buffer (Pierce, Thermo Fisher), the nuclei were removed by centrifugation, and the supernatant was mixed with 5× concentrated loading buffer (60 mM Tris, pH 6.8, 25% glycerol, 2% SDS, 5% β-mercaptoethanol, 0.1% bromophenol blue). The cell culture supernatants were directly mixed with 5× loading buffer. Samples were heat denatured (100°C, 5 min), centrifuged for 1 min at 13,000 rpm with a tabletop centrifuge (Eppendorf), and loaded into SDS-PAGE gels. The gels were run at 25 mA/gel for 1 h, and the separated proteins were subsequently transferred onto nitrocellulose membranes with a wet transfer system at 100 V for 1 h. The membranes were then blocked in 5% milk in PBS–0.1% Tween 20 (PBS-T) and incubated overnight with primary antibody. After 3 washes in PBS-T, the membranes were incubated with secondary antibody (IRDye labeled; LI-COR) in 5% milk for 1 h, washed again 3 times in PBS-T and 2 times in PBS, and detected using an Odyssey imaging system (LI-COR).

### Antibodies.

For the detection of tubulin β-III by immunofluorescence (IF), mouse monoclonal Tuji-1 antibodies were used (either Covance [catalog no. MMS-435P-0100; Eurogentec] antibodies at a 1:200 dilution or Millipore [catalog no. MAB5564] antibodies at a 1:300 dilution). The anti-SgG2 polyclonal rabbit serum was produced by Davids Biotechnologie GmbH (Regensburg, Germany) by twice injecting the peptides (LPPHWAPGALDDGPYAPFPPRPRFRR and EGPGPTAPPQAARAEGGPC) from SgG2 (the peptide sequences are based on previously published work [87]) in rabbits and collecting the sera at day 63 postinjection. This antibody was used at a 1:2,000 dilution in the Western blotting assays. To detect the MgG2 ectodomain, we used an anti-HSV-2 envelope antibody (catalog no. ab53470; Abcam). Anti-ICP5 (MCP) antibody (HSV-1/HSV-2) from Abcam (catalog no. ab6508) was used at a dilution of 1:1,000. Alexa Fluor fluorophore-coupled secondary antibodies (Invitrogen goat anti-mouse immunoglobulin-Alexa Fluor 488 [catalog no. A11029; Life Technologies]; donkey anti-mouse immunoglobulin-Alexa Fluor 647 [catalog no. A-31571; Thermo Fisher Scientific]) were used with these primary antibodies. To detect gG2 and gD in the cells infected with HSV-2 strain 333, we used a mouse monoclonal anti-gG2 antibody (catalog no. sc-69805; Santa Cruz) and a rabbit anti-gD serum (88) provided by Enrique Tabarés (Universidad Autónoma de Madrid, Madrid, Spain), respectively. These primary antibodies were detected with anti-mouse horseradish peroxidase (HRP)-IgG (1:5,000; catalog no. NXA931; GE Healthcare) and anti-rabbit HRP-IgG (1:5,000; catalog no. NA9340V; GE Healthcare), respectively. Detection was performed by enhanced chemiluminescence.

To neutralize NGF in neuronal cultures, we used mouse monoclonal anti-β-NGF (human) antibody from R&D Systems (catalog no. MAB2561) diluted 1:500 in medium.

### Culture of primary neurons from dissociated DRG.

Primary DRG neurons were dissociated from 35-day-old (±3 days) BALB/cJ-Ztm mice using a procedure adapted from previous work (31, 32). The mice were sacrificed, and the vertebral column was removed and longitudinally split with two sagittal cuts. The spinal cord was then removed to expose the DRG, which were then pulled out, and the attached large axonal processes were trimmed. The DRG were collected in neuron medium (DMEM and Ham’s F-12 medium supplemented with 10% FCS, Pen-Strep, and 50 ng/ml mouse NGF 2.5S [catalog no. G514A; Promega]) and then dissociated by sequential digestions with papain (20 min) and a dispase-collagenase mix (20 min). This was followed by disruption with 3 fire-narrowed Pasteur pipettes with decreasing diameters. The cells were washed and, finally, resuspended in 5 μl neuron medium per MFC (∼1 animal for 4 MFC). The cells were seeded into the somal compartment (SC) of non-plasma-bound MFC with 450-μm microgrooves (catalog no. RD450; Xona Microfluidics LLC, USA) according to the instructions in the manufacturer’s manual, with the exception that 0.1 mg/ml poly-l-lysine (catalog no. P4832; Sigma) was used to coat the coverslips (24 by 32 mm; Roth), followed by coating with laminin overnight (30 μl/MFC with 100 ng/μl). The leftover cell suspension was used to count the number of seeded cells (which was usually about 3 × 10^4^ cells/compartment but ranged from 2 × 10^4^ to 5 × 10^4^ cells/compartment). Since we always added the same volume per experiment, we had an equal number of cells per condition in each experiment. The cells were incubated for 24 h before changing the medium to neuron medium supplemented with aphidicolin (4 μg/ml; catalog no. A0781-1MG; Sigma) to inhibit the mitosis of nonneuronal cells. Note that aphidicolin also reduces the replication of DNA viruses. The neurons were then cultured for 3 days, if not stated differently.

### Coculture of neurons and HEK-293T cells and neurite recruitment assay.

Dissociated neurons were prepared and seeded as described above. HEK-293T cells were added to the neurite compartment at 24 h postseeding of the dissociated DRG cells. To do so, HEK-293T cells were scraped off 10-cm cell culture dishes (at 24 h postinfection [hpi]), pelleted, and resuspended in medium (∼150 μl). Ten microliters of this concentrated cell suspension was then added to the top well of the neurite compartment (NC) part of the MFC, containing the same amount of neuron medium (with NGF and aphidicolin) as the somal compartment (SC), resulting in an 80% to 90% confluent cell monolayer. The MFC were then incubated for 3 days, before the cells in the MFC were fixed and stained.

For the infection experiments, the cells were infected at an MOI of 0.1 in 1 ml (6-well format) and harvested using a cell scraper at 24 hpi, as described above. The number of infected cells was estimated by counting the mCherry-positive cells under a microscope. The infected cell suspension was then mixed with noninfected cells to produce a cell suspension with 20% mCherry-positive cells. Twenty microliters of this cell suspension was subsequently seeded into the MFC, as described above. This corresponded to approximately 2 × 10^5^ cells. For transcomplementation experiments, cells were transfected with 0.5 μg of the corresponding plasmids using the Lipofectamine 2000 reagent, as recommended by the manufacturer, on the same day that the neurons were prepared. The cells were washed with PBS at 4 h posttransfection and then infected at an MOI of 0.1. The treated cells were then harvested by scraping at 24 hpi and seeded in MFC, as described above.

For the neutralization of NGF, the medium of the neuronal cultures was changed at 24 h postseeding to medium containing 50 ng/ml NGF plus aphidicolin and 1:500-diluted anti-NGF antibody AB-09 (catalog no. MAB2561; R&D Systems), according to the manufacturer’s instructions. Infected HEK-293T cells were collected and concentrated as described above, anti-NGF antibody AB-09 was added to the cell suspension (1:500), and the cells were incubated for 5 min before they were added to the MFC. The cultures were then incubated for a further 2 days before fixing and IF staining.

### Immunofluorescence.

Immunofluorescence staining of cells in MFC was performed as recommended by the manufacturer (Xona manual, non-plasma-bound devices). In short, cells were fixed using 3% paraformaldehyde at room temperature for 30 min and then washed with PBS, permeabilized using 0.2% Triton X-100 for 30 min, and then blocked with PBS plus 3% bovine serum albumin (IF grade, IgG free) and 5% goat or donkey serum, depending on the secondary antibodies used, for at least 1 h. The primary antibodies were diluted in blocking solution as stated above, and then a pressure gradient was created by adding 50 μl to the NC and 100 μl to the SC. The MFC were then incubated with the primary antibodies at 4°C overnight. On the next day, the MFC were washed with PBS 3 times and incubated with secondary antibodies (diluted 1:1,000) in blocking solution for at least 2 h. After the incubation, the MFC were washed again and 2 drops of ProLong antifade reagent were added to the top SC well and 1 drop was added to the top NC well. The MFC were then incubated at 4°C overnight to allow the antifade reagent to reach into the microgrooves. The MFC were imaged on the next day using a Zeiss Observer Z1 inverted microscope.

### Quantification of neurite outgrowth.

To quantify neurite outgrowth, we used the Simple Neurite Tracer (47) plug-in in FIJI software (version 1.5 and higher). In short, multidimensional microscopic images from the Zeiss Observer microscope (Zeiss ECPlan Neofluar 10× objective; numerical aperture, 0.3 [∞/−]; catalog number 440330-9902) were loaded into the FIJI software and split into single-channel images, and the channel corresponding to tubulin β-III staining (Tuj1 antibody) was used for measuring neurites. Neurites were measured from the growth cone to their exit point from the microgroove, when possible, by following them and marking them with a path (count mask). To estimate the overall neurite density, all neurites visible in an image were covered with paths, including partially visible neurites. When forks in the path of a neurite were encountered, the shorter connection to the entry point was usually preferred; also, turning angles of more than 40° were avoided, unless there was a clear path. All endpoints were connected to the entry points, and any remaining neurites were measured as visible and connected to the closest entry point. To estimate neurite density, all marked paths were then measured by the plug-in, recording the number of paths and the length of every path.

### Statistical analysis.

For statistical analysis, the software GraphPad Prism (version 8.4.3 for Windows; GraphPad Software, San Diego, CA, USA) was used. We followed the recommendations of the journals *Journal of Virology* (89) and *Infection and Immunity* (90) regarding statistical analysis. Data on the neurite length and the number of neurites showed a lognormal distribution. Therefore, all data sets containing neurite length or the number of neurites were log_2_ transformed to make the data more normally distributed. Since in some experiments the data groups also showed a significant difference in variances (Bartlett’s test for equal variances) and the number of measurements (*n*) was different in many groups, several prerequisites for application of the standard one-way analysis of variance (ANOVA) were violated. Therefore, for all statistical analyses involving data sets with significantly different standard deviations (Bartlett’s test and the Browne-Forsythe test), a Welch/Browne-Forsythe-corrected ANOVA was used with an appropriate posttest, depending on the number (Games-Howell’s posttest for *n* > 50; Dunnett’s T3 posttest for *n* < 50). For data sets with equal variances, an ANOVA with Sidak’s posttest was applied. If the normality of the transformed data set could not be guaranteed (Fig. 5A), a nonparametric Kruskal-Wallis test was used. The results of Dunn’s posttest for multiple comparisons of selected columns and medians with interquartile ranges are displayed in this case. Adjusted *P* values for multiple testing, as reported by the Prism software, are indicated in the figures.

### Data availability.

The sequences have been uploaded into GenBank (accession no. MH796783 and MH796784) and the European Nucleotide Archive (accession no. ERS3367584 [https://www.ebi.ac.uk/ena/browser/view/ERS3367584] and ERS3367585 [https://www.ebi.ac.uk/ena/browser/view/ERS3367585]).
